# Rebuilding Working Lives After Forced Migration: Career Reconstruction as Acculturative Adaptation Among Syrian Migrants in Türkiye

**DOI:** 10.3390/bs16101835

**Published:** 2026-10-08

**Authors:** Ahmet Ayaz, Özgür Osman Demir, Fevzeddin Gök

**Affiliations:** 1 Department of Guidance and Psychological Counseling, Faculty of Education, Hasan Kalyoncu University, Gaziantep 27000, Türkiye; ozgur.demir@hku.edu.tr; 2 Presidency of the Republic of Türkiye Presidency of Religious Affairs, Şanlıurfa 63000, Türkiye; fevzeddin.gok@std.hku.edu.tr

**Keywords:** forced migration, acculturation, career reconstruction, Syrian migrants, social support, migrant agency

## Abstract

Forced migration disrupts occupational trajectories, professional identities and processes of acculturative adaptation. Yet less is known about how displaced people rebuild working lives while navigating host-society legal, linguistic, economic and sociocultural conditions. This exploratory qualitative descriptive study examines career reconstruction as a domain-specific form of acculturative adaptation among Syrian migrants in Türkiye. Semi-structured interviews were conducted with ten participants, five women and five men, who had worked before migration and experienced occupational change after resettlement. Content analysis identified two interrelated dimensions. First, participants described barriers linked to limited institutional guidance, language barriers, legal constraints, financial pressure, perceived loss of agency and gendered responsibilities. Second, they mobilised informal social support, self-efficacy, future orientation, occupational exploration and transferable professional skills to navigate precarious labour-market conditions. The findings show that post-migration career trajectories are not simply linear stories of decline or survival, but negotiated processes shaped by the interaction between structural inequality, social networks and migrant agency. The article contributes to acculturation and migration scholarship by conceptualising work and occupational identity as central sites of social and psychological adaptation after forced migration. It also highlights the need for rights-based, culturally responsive and community-linked support for labour-market integration.

## 1. Introduction

Migration is an enduring social phenomenon that has shaped human history and continues to constitute a shared global reality (Bartram et al., 2014). Although migration may occur voluntarily or involuntarily and may involve movement within or across national borders, contemporary migration flows are increasingly shaped by political instability, armed conflict, persecution and disasters (International Organization for Migration, 2019; Sirkeci & Cohen, 2015). In this context, forced migration refers to situations in which individuals are compelled to leave their places of residence against their will because of conflict, violence, political disputes or disasters (Migration Data Portal, 2024). Globally, the number of international migrants has reached approximately 280 million, while the number of forcibly displaced individuals has risen to 122 million (UNHCR, 2025). These figures indicate that forced migration is not only a demographic or humanitarian issue but also a multidimensional social and psychosocial process that affects identity, belonging, social integration, economic participation and future life trajectories.

Individuals who experience forced migration are often required to rebuild their lives under conditions of uncertainty and structural constraint. Previous studies have shown that forcibly displaced populations commonly face housing insecurity, health and nutrition problems, psychological distress, educational disruption, low literacy and limited proficiency in the host country’s language (Aslan & Güngör, 2019; Demir & Aliyev, 2019; Erdoğan, 2020; UNHCR, 2025). These challenges are not isolated; rather, they interact with one another and shape migrants’ opportunities for participation in education, employment and social life. Among these challenges, work-related difficulties are particularly critical because employment is closely connected to economic survival, social recognition, identity continuity and psychological well-being. Forced migration may therefore disrupt not only where people live but also who they are professionally and how they imagine their futures.

Career development among forcibly displaced individuals is often marked by discontinuity, occupational mismatch, deskilling and loss of professional status. Migrants who previously worked as teachers, nurses, engineers, drivers or business owners may be unable to continue their pre-migration occupations because of language barriers, legal restrictions, non-recognition of qualifications, limited social networks and precarious labour-market conditions (Arthur et al., 2025; Bauder, 2003; Guo, 2009; Sultana, 2022; Visintin et al., 2015). As a result, many are compelled to enter low-status, informal or survival-oriented jobs that do not correspond to their prior education, competencies or occupational identities. This process may produce feelings of injustice, diminished self-worth and reduced career agency, while also complicating broader social integration (Ager & Strang, 2008; Morici et al., 2022; Pajic et al., 2018).

The occupational changes experienced by migrants after displacement can be conceptualised as career transitions. Career transition refers to movement from familiar occupational roles to new work roles that may differ substantially from one’s previous career trajectory (Latack, 1984). In forced migration contexts, however, career transition is rarely a planned or voluntary process. Rather than being driven primarily by personal choice or career advancement, it often emerges from disruption, necessity and adaptation to restrictive conditions. Heppner’s (1998) classification of career transitions as task change, position change and occupational change is useful in this regard, because many forcibly displaced individuals experience not merely a change in task or position but a more radical occupational change that requires them to reconstruct their working lives in unfamiliar social, cultural and institutional environments.

Understanding career transitions after forced migration requires a theoretical perspective that considers both individual meaning-making and structural conditions. Career Construction Theory explains how individuals reconstruct career narratives and professional identities in response to major life transitions and contextual disruption (Savickas, 2005, 2013). Social Cognitive Career Theory further helps explain how self-efficacy, perceived barriers and agency shape career exploration and decision-making under uncertainty (Lent et al., 1994; Lent & Brown, 2013). However, forced migrants’ occupational pathways cannot be explained solely through individual psychological resources. Ecological and systems-based perspectives emphasise that career development is embedded within interacting social, cultural, legal, economic and institutional systems (Bronfenbrenner, 1979; W. Patton & McMahon, 2014), while Social Identity Theory and Self-Categorisation Theory highlight the role of belonging, group membership and perceived exclusion in migrants’ work-related experiences (Tajfel & Turner, 1979; Turner et al., 1987). Together, these perspectives frame career reconstruction as a dynamic process shaped by structural constraints, migrant agency and available resources.

From an acculturation perspective, the labour market is not only an economic setting but also a key arena in which social and psychological adaptation unfolds. Acculturation and adaptation perspectives suggest that migrants negotiate new cultural expectations, institutional norms, language practices and social relationships while maintaining links to prior identities and developing competencies for participation in the host society (Berry, 1997; Ward & Geeraert, 2016; Ward & Kennedy, 1999). In this study, career reconstruction is therefore understood as a domain-specific form of acculturative adaptation. Syrian migrants’ attempts to rebuild working lives are examined as part of their broader efforts to navigate host-society legal procedures, workplace expectations, linguistic demands, community networks and changing forms of belonging. This framing specifies a contribution to acculturation theory: work is a setting in which access to host-society institutions, recognition of prior skills and continuity of occupational identity are negotiated together. Employment status alone cannot capture these processes, because adaptation also depends on how migrants interpret occupational change and act within available opportunities.

Türkiye offers an important context for examining these issues. Over the past decade, Türkiye has become one of the major destination countries for large-scale forced migration, particularly from Syria. As of 2025, approximately 2.4 million Syrians are living in Türkiye under temporary protection, and a substantial proportion of this population is of working age (Directorate General of Migration Management, 2025). However, Syrians’ participation in the Turkish labour market often occurs under informal, low-wage and precarious conditions. Existing studies indicate that migrants’ educational backgrounds and professional qualifications are frequently not recognised, and many are unable to continue in their pre-migration occupations because of language barriers, legal status uncertainty and structural obstacles in the labour market (Canbey-Özgüler, 2018; Karasu, 2016; UNHCR, 2023; Yıldırımalp et al., 2017).

The temporary protection regime provides a legal framework for Syrians’ stay in Türkiye, yet access to formal employment remains shaped by work permit procedures, employer practices and informal labour-market dynamics. At the time of the May–June 2024 interviews, formal wage employment for Syrians under temporary protection generally required an employer-initiated work-permit application and was linked to the province of registration; informal employment remained another route into the labour market (Tumen, 2023). In addition to Turkish-language demands, access to jobs could depend on kinship and community contacts: research with Syrian women working in Istanbul found that relatives and neighbours helped participants obtain work in informal textile workshops (Gazioglu Terzi & Korkmaz, 2022). Care responsibilities and family expectations could further restrict women’s participation in training and paid work (CARE International, 2020). The coexistence of long-term residence and a temporary protection framework makes Türkiye particularly informative for examining how migrants pursue occupational continuity while navigating work-related procedures, language demands and informal employment networks over time.

Although previous research has examined migrants’ labour-market integration, social adaptation and employment barriers (Ager & Strang, 2008; Sultana, 2022; Yıldırımalp et al., 2017), less is known about how work-related experiences function as part of acculturative adaptation after forced migration. Acculturation research has often focused on language learning, cultural orientation, social belonging, psychological adjustment and intergroup relations (Berry, 1997; Ward & Geeraert, 2016; Ward & Kennedy, 1999). However, the reconstruction of working life remains an important but relatively underexamined domain in which social and psychological determinants of adaptation become visible. For forcibly displaced individuals, rebuilding a career may involve more than finding employment; it may also require negotiating legal uncertainty, language barriers, financial hardship, gendered expectations, social networks, self-efficacy, perceived agency, future orientation and the meaning of prior occupational identities. Addressing this gap is important for understanding how displaced people rebuild not only economic participation but also identity, belonging and future direction in the host-society context.

Against this background, the present study examines how Syrian migrants in Türkiye reconstruct their working lives after forced migration. Rather than treating employment as a narrow indicator of integration or career transition as a simple movement from one occupation to another, the study conceptualises career reconstruction as a domain-specific form of acculturative adaptation shaped by structural constraints and social and psychological resources. Accordingly, the study was guided by two research questions: What structural, social and psychological conditions shape the career reconstruction processes of Syrian migrants after forced migration to Türkiye? What personal and social resources do migrants draw upon while reconstructing their careers as part of acculturative adaptation in the host-society context? By addressing these questions, the study contributes to acculturation, migration and career scholarship by showing how displaced people reinterpret, negotiate and rebuild occupational identities under unequal legal, linguistic, economic and sociocultural conditions.

## 2. Method

### 2.1. Study Design

This exploratory study employed a qualitative descriptive design to examine how Syrian migrants described changes in their working lives after forced migration. This design was appropriate because the study sought to provide a context-sensitive account of participants’ experiences while remaining close to their meanings and narratives (Sandelowski, 2000; Kim et al., 2017). The study was situated within an interpretivist–constructivist paradigm, which understands experience through meanings constructed within social, cultural and institutional contexts (Creswell, 2013; Lincoln & Guba, 1985). Semi-structured interviews elicited accounts of occupational disruption, structural barriers, available resources and changes in occupational identity. Qualitative content analysis was used to identify recurring meanings, categories and differences across participants’ accounts.

### 2.2. Participants and Sampling

Participants were selected through criterion sampling, a purposive strategy used to identify information-rich cases with direct experience of the phenomenon under investigation (M. Q. Patton, 2015). The inclusion criteria were being a Syrian adult who had experienced forced migration to Türkiye, having migrated at least five years before the study, having worked before migration and being engaged in paid work in Türkiye after occupational change. These criteria ensured that participants could reflect on both pre- and post-migration occupational trajectories.

The study group consisted of ten Syrian migrants, five women and five men. Their sociodemographic and occupational characteristics are presented in Table 1. Participants were aged between 27 and 56, and their educational backgrounds ranged from no formal education to bachelor’s degree. Their pre-migration occupations included professional, skilled, service and manual work, while their post-migration jobs reflected varying degrees of occupational change, including downward mobility, partial transfer of previous skills, survival-oriented employment and attempts to rebuild professional identity. For example, participants had previously worked as teachers, an architect, a nurse, a construction worker, a shop owner and drivers, while their current work included barbering, farming, accounting, cleaning, tailoring and animal care. This occupational and educational variation helps illuminate different forms of skill transfer and occupational mismatch within the purposively selected sample.

All participants had migrated to Türkiye between 2013 and 2016 and had therefore lived in the host country for a substantial period before the interviews. This enabled the study to move beyond immediate survival experiences and examine accumulated encounters with the Turkish labour market, language demands, legal procedures and sociocultural environment. Variation in gender, age, education and occupational background further supported exploration of how career reconstruction was shaped by structural constraints and personal and social resources. Participants self-reported their gender as part of the personal information form. Because the study focused on socially patterned responsibilities, family expectations and labour-market experiences, the term gender is used when interpreting caregiving, mobility and occupational constraints.

The ten participants were purposively selected because they could describe occupational change before and after forced migration. Recurring patterns relevant to the research questions were observed across their accounts, and no new main thematic domain was identified in the final interviews. Given the sample size and the single interview with each participant, this observation is limited to the present dataset and should not be taken as evidence that the full range of relevant experiences was captured.

### 2.3. Data Collection Materials

A researcher-developed personal information form was used to collect sociodemographic and migration-related information, including gender, age, educational background, marital status, place of origin, year of migration, and pre- and post-migration occupations. These data helped contextualise participants’ occupational trajectories, skill transfer, gendered responsibilities and length of residence in Türkiye.

Data were collected using a semi-structured interview protocol developed by the research team and informed by literature on forced migration, acculturation and sociocultural adaptation, career transitions, migrant labour-market integration, occupational identity and support needs. The semi-structured format ensured that all participants were asked about core aspects of post-migration career reconstruction while allowing them to elaborate on personally meaningful experiences.

The protocol was organised around three domains: pre-migration occupational life, the migration process and post-migration career reconstruction. These domains explored participants’ educational and occupational histories, migration-related disruption, occupational change after resettlement, perceived barriers, support resources, use of previous skills and future work expectations.

The initial question pool was reviewed by three academics: two with expertise in qualitative research and career counselling and one with experience in qualitative research with migrant populations. Feedback focused on clarity, cultural appropriateness, sensitivity and relevance to the research questions. Based on this feedback, overlapping questions were removed, wording was refined and additional probes were added.

Before the main interviews, two pilot interviews were conducted with Syrian migrants who met the inclusion criteria but were not included in the final sample. The pilot interviews indicated that the questions were understandable and culturally appropriate, and minor wording changes were made to improve clarity and conversational flow. The final protocol included nine main questions and follow-up probes. Example questions included: “What was your working life like before migration?”, “How would you describe your occupational trajectory after migration?” and “What factors shaped your choice of current work?”.

### 2.4. Data Collection

Data collection began after ethics committee approval had been obtained. Participants were reached through personal contacts and community-based social networks in areas with a high concentration of Syrian migrants. Interviews were conducted face to face between May and June 2024, at times and locations agreed with participants and suitable for privacy, minimal noise and comfort. Before the interviews, participants were provided with an informed consent script that explained the purpose of the study, the voluntary nature of participation, confidentiality procedures, audio recording, the right to withdraw at any stage, and the use of anonymized quotations. The researcher also explained these points orally before each interview. Participants signed the informed consent form before the interviews, including providing permission for audio recording. One interview lasted approximately 25 min, while the remaining nine lasted approximately 40–45 min. All interviews were audio-recorded with participants’ consent. Interviews were conducted in Arabic by a native Arabic-speaking researcher with native-level Turkish proficiency. The interviewer used probing questions to explore participants’ answers in greater depth. In response to peer review, validation interviews were conducted by telephone in September 2026 with the two participants who could be reached and agreed to take part (P3 and P6). Of the remaining eight participants, five could not be reached and three were reached but declined a further interview. The accounts of P3 and P6 represented contrasting occupational paths: partial skill transfer and starting over after leaving a profession. The interviews lasted approximately 26 min (P3) and 22 min (P6), were conducted in Arabic by the original interviewer after verbal consent was obtained, and were documented through detailed notes taken during the calls.

Audio recordings were transcribed verbatim in Arabic and then translated by a bilingual Arabic–Turkish researcher. During translation, attention was paid to preserving participants’ intended meanings, culturally specific expressions and work-related terminology. To protect confidentiality, identifying information was removed from the transcripts, and participants were assigned codes such as P1, P2 and P3. Audio recordings and transcripts were stored securely and were accessible only to the research team.

### 2.5. Data Analysis

Qualitative content analysis was used to examine participants’ narratives and identify recurring meanings, categories and themes related to post-migration career reconstruction. The analysis was guided by Merriam’s (2009) qualitative analysis framework and supported by MAXQDA 24. This approach was appropriate because the study aimed to organise participants’ accounts of occupational barriers and social and psychological resources systematically while remaining close to their contextual meanings. Since the interviews were conducted in Arabic and translated into Turkish, the analysis was carried out using the translated transcripts, while attention was paid to preserving participants’ intended meanings, culturally specific expressions and work-related terminology.

The analysis proceeded in several stages. First, all researchers read the translated transcripts repeatedly to become familiar with the data and discuss initial impressions. A subset of interviews was then coded collaboratively to develop a shared understanding of the coding process and refine preliminary codes. Two researchers subsequently coded the remaining transcripts independently. Coding decisions were compared in joint meetings, and differences were discussed until consensus was reached. Codes with similar meanings were grouped into subcategories and broader categories, and the research team examined relationships among codes, subcategories and categories to develop the main thematic domains. Coding consistency was strengthened through independent coding, repeated comparison, consensus meetings and researcher triangulation. In the final stage, all researchers reviewed the analytic structure together and agreed on the final themes. To enhance coherence and credibility, two academics with expertise in qualitative research and career counselling reviewed the coding structure and thematic organisation. Their feedback focused on the clarity, relevance and internal consistency of the themes, and the presentation of the findings was refined accordingly.

### 2.6. Researcher Positionality and Reflexivity

Given the interpretive nature of the study and the sensitivity of researching forced migration, researcher positionality and reflexivity were considered throughout the research process. The research team consisted of academics and practitioners with backgrounds in psychological counselling, career counselling and qualitative research, and had longstanding experience working with migrant populations. These backgrounds provided familiarity with career development, psychosocial adaptation and counselling-related issues, but also required the researchers to remain attentive to the possibility of interpreting participants’ narratives through pre-existing professional and disciplinary assumptions. The researchers were also aware that differences in educational background, professional status and researcher–participant roles could create power asymmetries during the interviews. To reduce these effects, attention was paid to creating a respectful interview environment in which participants could decide how much they wished to share and how they wished to describe their experiences.

Reflexivity was maintained through regular discussions during data collection and analysis. In these meetings, the researchers examined their assumptions and initial interpretations regarding forced migration, career development and migrants’ labour-market participation. Analytic decisions were not made by a single researcher; instead, collaborative reading, independent coding, consensus meetings and final review by the research team were used to question individual interpretations and strengthen analytic coherence. These strategies helped the researchers remain close to participants’ accounts while critically considering how their own professional positions, disciplinary backgrounds and assumptions might shape the interpretation of the data.

### 2.7. Trustworthiness

Several strategies were used to enhance the trustworthiness of the study. Credibility was supported through repeated engagement with the transcripts, collaborative and independent coding, consensus meetings and researcher triangulation. A summary of the main interpretations was also shared with participants to assess whether the findings resonated with their experiences (Merriam, 2009). In addition, two external academics with expertise in qualitative research and career counselling reviewed the coding structure and thematic organisation in terms of coherence, clarity, relevance and internal consistency. Transferability was addressed through thick description by presenting participants’ sociodemographic characteristics, migration histories, pre- and post-migration occupations and the Turkish migration context, allowing readers to assess the applicability of the findings to similar contexts (Geertz, 1973; Ponterotto, 2006). Direct quotations were also used in Section 3 to provide contextualised evidence for the themes and preserve participants’ voices.

In addition, the two validation interviews offered a direct cross-check of the interpretations. The interviewer read a plain-language summary of the main findings and asked participants whether it reflected their experiences, which barriers and resources had been most important to them, whether anything important was missing, and how their current work related to their previous occupation. Both participants recognised the findings most closely related to their own experiences and elaborated on them; they described the remaining findings as familiar from the experiences of other Syrian migrants they knew. Neither participant disputed any of the findings, and their comments reinforced rather than altered the interpretations. Because participants may be inclined to agree with researchers’ summaries, we treat these interviews as a check on the resonance of the interpretations rather than as proof of their accuracy (Birt et al., 2016).

Dependability was enhanced by systematically documenting the research process, including participant recruitment, interview procedures, transcription, translation, coding, category development and theme refinement. Confirmability was supported by maintaining an audit trail consisting of anonymised transcripts, translated materials, coding schemes, analytic notes and records of decisions made during consensus meetings (Lincoln & Guba, 1985; M. Q. Patton, 2015). Together, these procedures strengthened the transparency, coherence and credibility of the analytic process and helped ensure that the findings were grounded in participants’ narratives rather than in the assumptions of a single researcher.

The interview evidence was also appraised in light of Small and Calarco’s (2022) five indicators. Conducting interviews in Arabic and attending to participants’ own meanings supported cognitive empathy. Contrasting occupational paths and the mixed role of familiar networks illustrate heterogeneity. Quotations offer direct evidence but are often brief (palpability). The interviewer used responsive probing during interviews (follow-up), while team discussions of interpretations and researcher assumptions supported self-awareness. These practices do not replace prolonged exposure: each participant took part in one in-depth interview, and only two took part in a subsequent brief validation interview.

## 3. Findings

The analysis showed that participants’ post-migration working lives were shaped by a dynamic tension between structural constraints and social and psychological resources. Rather than a simple movement from one occupation to another, career reconstruction emerged as a domain-specific form of acculturative adaptation in which participants sought to maintain occupational continuity, respond to survival needs and rebuild professional identity within restrictive legal, linguistic, economic and sociocultural conditions. In line with the research questions, the findings are organised into two interrelated thematic domains: barriers to career reconstruction and resources supporting career reconstruction. The first captures the structural and host-society conditions that disrupted occupational continuity, while the second reflects the personal, social and cultural resources through which participants negotiated these constraints and pursued adaptation in working life.

### 3.1. Barriers to Career Reconstruction

**Figure 1:**
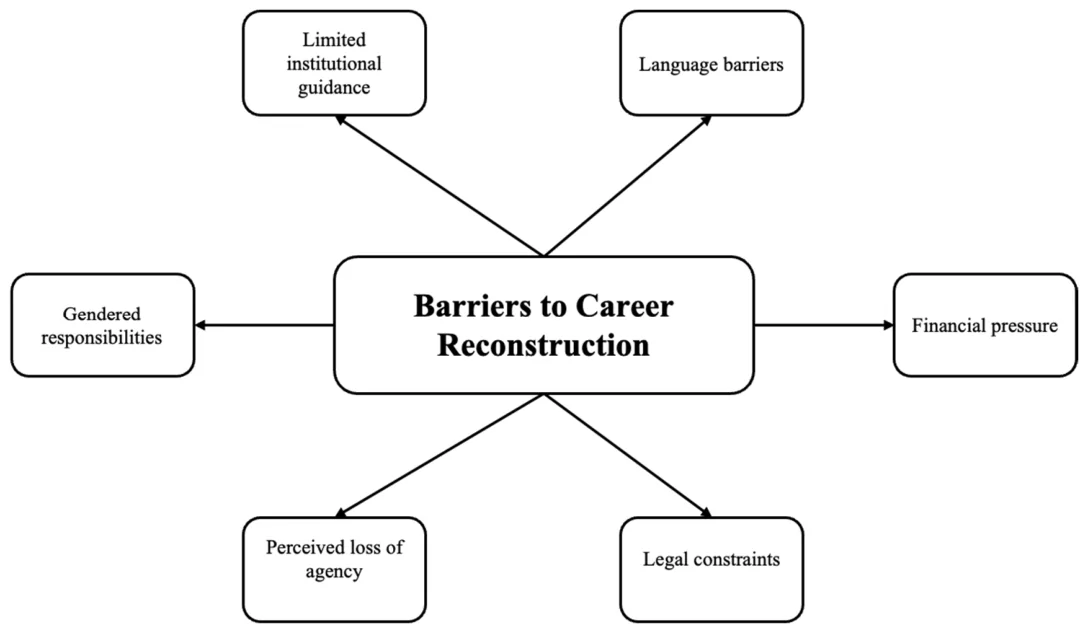
Barriers to career reconstruction among forced migrants.

Participants’ post-migration career reconstruction was constrained by interrelated barriers operating at structural, institutional, linguistic, economic and sociocultural levels. These barriers rarely appeared in isolation; rather, they shaped the conditions under which participants attempted to adapt to the host society through working life. Limited language proficiency restricted access to information and formal employment, financial hardship pushed participants towards immediate income-generating work, and gendered responsibilities narrowed women’s occupational choices. As shown in Figure 1, six main barriers were identified: limited institutional guidance, language barriers, legal constraints, financial pressure, perceived loss of agency and gendered responsibilities.

### 3.2. Limited Institutional Guidance

One of the most frequently mentioned barriers was the absence of systematic guidance in navigating the post-migration labour market. Participants reported uncertainty about available occupations, entry routes, legal procedures and training opportunities. This lack of information made occupational decision-making more difficult and often left participants dependent on informal advice from relatives or acquaintances. As P7 explained, “One of the main problems I experienced was not knowing what I could do in this country.” Similarly, P10 stated, “I did not know what to do. Fortunately, my relatives guided me.”

Participants’ accounts suggest that guidance needs extended beyond simple job placement. They needed support in understanding the labour market, identifying transferable skills, accessing training and interpreting institutional procedures. Some participants also described available support as difficult to use in practice. For example, P6 stated, “UNICEF offered an opportunity, but it was a long-term training program, and I could not leave my children alone.” This illustrates how support mechanisms may remain inaccessible when they do not align with migrants’ caregiving responsibilities, financial pressures or everyday constraints.

### 3.3. Language Barriers

Language barriers were among the most visible obstacles to occupational continuity. Participants stated that limited Turkish proficiency prevented them from using their pre-migration qualifications and skills in Türkiye. P7 explained, “In order to continue working as a teacher in my field, I need to have a good command of Turkish.” P4 similarly noted, “Language is the main reason I cannot practice architecture.” These accounts show that language barriers affected not only communication but also professional identity, occupational status and access to qualified work.

Some participants developed coping strategies to reduce the impact of language barriers. For instance, P2 stated, “By choosing a region where Arabic is spoken, I partially overcame the barrier.” Such linguistic buffer zones provided short-term communicative ease and helped participants secure work or social support. However, they also risked limiting longer-term integration by confining participants to narrower social and economic networks.

### 3.4. Legal Constraints

Legal status and related procedures also shaped participants’ educational and occupational opportunities. Several participants associated their restricted access to education, employment or entrepreneurship with the absence of citizenship or uncertainty about legal rights. P2 stated, “Because I am not a citizen, there are many things I cannot do.” P1 similarly said, “In order for me to study, citizenship is required.” These statements should be read as participants’ perceptions of legal constraint rather than as a formal legal assessment.

Participants also reported limited knowledge about procedures related to business establishment, licensing, credential recognition and access to formal employment. This suggests that legal constraints were not only about formal rights, but also about migrants’ ability to understand and navigate institutional systems. In this sense, limited legal information contributed to a sense of uncertainty and restricted participants’ capacity to plan their occupational futures.

### 3.5. Financial Pressure

Financial hardship was another central barrier. Most participants described arriving in Türkiye with limited or no economic resources and needing to prioritise immediate survival over long-term career goals. P1 stated, “If you have no financial resources, you cannot do anything.” P2 similarly noted, “We arrived with nothing, and we had no margin for error.”

These accounts indicate that economic insecurity narrowed participants’ room for occupational choice. Under financial pressure, participants often accepted available jobs regardless of whether these jobs matched their previous education, skills or professional identities. Long-term plans such as further education, retraining or re-entering a previous profession were postponed because immediate income was necessary for family survival.

### 3.6. Perceived Loss of Agency

Some participants described their post-migration career trajectories as being shaped by circumstances beyond their control. These accounts were closely linked to war-related loss, age, family responsibility and limited opportunities. P6 explained, “I did not have time to think about my options; I lost my spouse and my son in the war, and I had to survive.” Her account shows how traumatic loss and urgent survival needs can restrict future-oriented planning.

Similarly, P7 stated, “I am 33 years old; going back to school and being appointed again feels impossible to me.” This statement reflects how participants’ perceptions of age, institutional barriers and requalification demands could reduce their sense of occupational possibility. Rather than indicating a lack of motivation, these narratives reveal how repeated constraints may weaken migrants’ perceived control over their career reconstruction.

### 3.7. Gendered Responsibilities

Women’s career reconstruction was particularly shaped by family responsibilities, childcare and culturally embedded gender expectations. Female participants described how caregiving duties limited their ability to participate in training, accept certain jobs or continue previous occupations. P6 stated, “I have children, and all of them are girls… I needed to stay close to my family, and my options became very limited.” Her account shows how post-migration labour-market participation was shaped not only by individual qualifications but also by family responsibilities and gendered expectations.

These gendered constraints intersected with other barriers such as financial pressure, limited institutional support and legal uncertainty. As a result, women’s occupational options were often narrowed more sharply, especially when available support systems did not take childcare and family obligations into account.

Taken together, these barriers show that participants’ career reconstruction was constrained by the cumulative interaction of legal, linguistic, economic, institutional and gendered conditions. These constraints limited access to education, formal employment and occupational continuity, while also shaping participants’ sense of possibility and control over their future working lives.

The two thematic domains were interconnected rather than successive stages. Limited institutional guidance often increased reliance on relatives and acquaintances, while language barriers led some participants to seek Arabic-speaking settings. Financial pressure could delay training or occupational plans even when participants expressed confidence or future goals. These examples show how available resources shaped responses to barriers without necessarily removing the constraints themselves.

### 3.8. Resources Supporting Career Reconstruction

**Figure 2:**
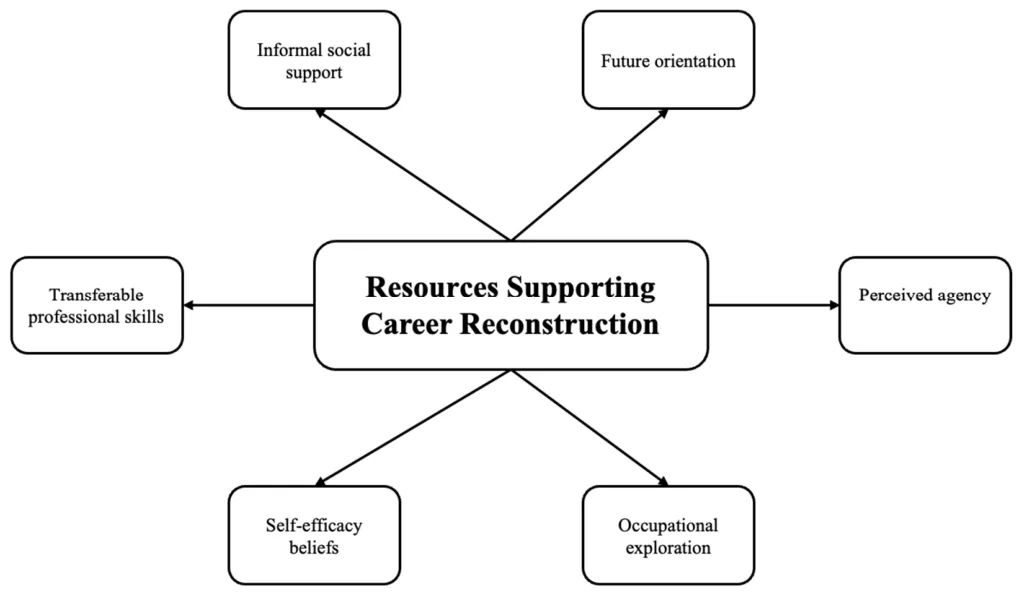
Resources supporting career reconstruction among forced migrants.

Resources supporting career reconstruction emerged as the social and psychological means through which participants attempted to negotiate, compensate for or partially overcome the barriers described above. These resources did not remove structural constraints, but they helped participants create limited spaces of action within restrictive conditions and supported their acculturative adaptation through working life. As shown in Figure 2, six resources were identified: informal social support, future orientation, perceived agency, occupational exploration, self-efficacy beliefs and transferable professional skills. Participants’ narratives show that post-migration career reconstruction was supported mainly through informal networks and personal resources, while formal institutional support remained limited or difficult to access.

### 3.9. Informal Social Support

Informal social support was one of the most important resources in participants’ post-migration occupational trajectories. In the absence of systematic institutional guidance, relatives, friends, local residents and migrant community networks provided practical help, information and access to work opportunities. P1 described how local support enabled him to start working: “I opened a shop in an empty room in the village; a benefactor provided it.” Similarly, P10 explained, “They picked us up from the border, opened their homes to us, and together we explored what kind of work I could do.”

Social support also provided emotional security and a sense of belonging. Participants felt more able to adapt in places where they encountered linguistic and cultural familiarity. P2 stated, “When I saw people speaking Arabic where I went, I felt relieved and became more committed to my work.” P9 similarly noted, “I came to Urfa and did not feel like a stranger at all.” These accounts suggest that culturally familiar environments could function as short-term buffers, helping participants access social ties and work-related information.

For some participants, immediate social circles directly shaped occupational choices. P7 explained, “People around me said I could work as an interpreter in a touristic area, and I became excited.” Family members also contributed to women’s work trajectories. P9 stated, “My daughters supported me in my work.” However, such support was mostly informal and unevenly available, meaning that it could assist career reconstruction but could not fully compensate for the absence of formal guidance.

### 3.10. Future Orientation

Despite the uncertainty created by forced migration, some participants maintained future-oriented goals. Future orientation helped them preserve hope, reinterpret previous occupational identities and imagine possible career pathways. P1 linked his current work to future plans: “By returning to education, I can make sure that my previous efforts were not in vain. God willing, I can succeed. I also have goals in barbering—to expand my shop, move to a more developed city, and work there.”

Participants also connected future planning with personal interests and contextual realities. P9 explained that she chose her current occupation by considering both her circumstances and preferences: “I needed a new beginning and a new plan… I also enjoy working with animals, so I chose this job.” Similarly, P7 stated, “My new job gave me the same feeling I had when I was a teacher, and I began to look more positively toward the future.” These narratives show that forced migration did not entirely eliminate future planning; rather, participants sought to build new possibilities within constrained conditions.

### 3.11. Perceived Agency

Some participants described active efforts to shape their post-migration occupational pathways despite structural barriers. P4’s account illustrates this sense of agency: “I did not want to give up. First, I took the open education and YÖS exams here… After passing these exams, I was admitted to the accounting department.” Her narrative shows how educational re-entry and acceptance of a new work opportunity became part of career reconstruction.

P6 also described a difficult but active process of rebuilding after leaving nursing: “I left nursing… I felt a sense of emptiness. I left a job I had devoted my life to and started everything from scratch. This was my career, and I wanted to succeed—and I did.” These accounts suggest that perceived agency enabled some participants to make decisions, persist through uncertainty and reinterpret occupational change as a process of rebuilding rather than only loss.

### 3.12. Occupational Exploration

Participants also engaged in occupational exploration by searching for information, observing others and considering alternative work pathways. P5 described seeking information online: “I tried to shape my career on my own by looking things up on the internet.” Similarly, P3 stated, “I was constantly researching, thinking about what I could do.”

For some participants, exploration remained at the level of future possibility rather than immediate action. P9 explained that she hoped to open a grocery store after saving money but still needed to learn about the required procedures and permits. This shows that exploration varied across participants: some moved from inquiry to action, while others remained in a planning stage because of financial, legal or family-related constraints.

### 3.13. Self-Efficacy Beliefs

Self-efficacy beliefs supported participants’ motivation to pursue new occupational goals. P1 explained how observation and learning strengthened his confidence: “I observed people around me who were doing this job, and once I understood it better, I believed that I could do it as well.” His account suggests that confidence developed not in isolation but through exposure to others’ work and gradual familiarisation with the new occupation.

Other participants connected self-efficacy to long-term occupational aspirations. P5 stated, “My dream of opening my own school will never fade… after nearly 20 years of experience in this field, the likelihood of failure is very low.” P8 similarly expressed confidence in expanding his current work with animals and animal-based production. These narratives indicate that self-efficacy helped participants sustain motivation and imagine occupational futures beyond immediate survival.

### 3.14. Transferable Professional Skills

Participants also drew on skills and knowledge acquired before migration. For some, previous occupational experience provided continuity between pre- and post-migration work. P3 explained, “The profession I had before migration and the one I am doing now are very similar—almost the same. In one I was operating an excavator, and in the other I am operating a tractor.” This continuity made adaptation to the new occupation easier.

Other participants transferred skills more indirectly. P4 noted, “They are not very close, but there are similarities. There is also accounting in architecture, and this made my current job easier.” Such accounts show that prior professional skills could act as bridges between disrupted occupational identities and new forms of work, even when participants could not continue in their original professions.

Taken together, these resources helped participants preserve continuity, motivation and a sense of agency within constrained post-migration conditions. Informal social networks compensated partly for limited institutional guidance, while transferable skills connected past and present occupational identities. Future orientation, perceived agency, occupational exploration and self-efficacy enabled participants to imagine and pursue possible career pathways. However, these resources were unevenly distributed and remained shaped by gender, education, previous occupation and access to supportive networks.

### 3.15. Integrated Summary of Findings

Overall, the findings show that post-migration career reconstruction was shaped by the interaction between structural constraints and social and psychological resources. Structural barriers such as limited institutional guidance, language barriers, legal constraints, financial pressure, gendered responsibilities and perceived loss of agency constrained participants’ occupational options and disrupted continuity in their working lives. At the same time, resources including informal social support, future orientation, perceived agency, occupational exploration, self-efficacy beliefs and transferable professional skills helped participants negotiate these constraints and pursue new occupational pathways.

The findings suggest that forced migration disrupted occupational continuity and professional identity by placing individuals within restrictive legal, linguistic, economic and sociocultural conditions. These conditions often resulted in occupational mismatch, deskilling, reduced room for manoeuvre and survival-oriented work. However, participants did not respond to these disruptions in identical ways. They drew on available social networks and personal resources to reconstruct their careers within the opportunities and limitations of the host context. Career reconstruction therefore emerged as a negotiated form of acculturative adaptation shaped by the interaction between structural inequality, social support and migrant agency.

Taken together, the findings indicate that post-migration career trajectories were neither linear nor defined solely by occupational decline. Rather, they reflected adaptive and negotiated processes in which displaced individuals sought to balance systemic barriers with available resources while rebuilding occupational continuity, professional meaning and future direction. In this sense, working life operated as a key domain in which social and psychological determinants of acculturation became visible. For example, P7 linked difficulty returning to teaching to Turkish-language demands, P3 described transferring machine-operation skills to tractor work, and P6 described rebuilding after leaving nursing. These accounts illustrate blocked occupational continuity, partial skill transfer and starting over within the same broad process of career reconstruction.

## 4. Discussion

This study examined how Syrian migrants in Türkiye reconstructed their working lives after forced migration. The findings show that post-migration career reconstruction was neither a linear occupational transition nor a purely individual process of adaptation. Rather, it emerged through the interaction between structural constraints, including limited institutional guidance, language barriers, legal constraints, financial pressure and gendered responsibilities, and personal and social resources such as informal support, perceived agency, self-efficacy, occupational exploration, future orientation and transferable professional skills. Barriers and resources similar to those identified here have been discussed in research on refugees’ career development and labour-market integration (Beqiraj & Ferrari, 2023; Sultana, 2022). Chang (2025) has also examined occupational change and continuity among Syrians in Türkiye. The contribution of the present study is therefore a bounded interpretation of how employment, the use of prior skills and continuity of occupational identity related to one another in the accounts of ten employed migrants who had changed occupations. P3 described transferring machine-operation skills to tractor work, whereas P6 described a sense of emptiness after leaving nursing and an effort to rebuild working life in tailoring. These accounts illustrate how being employed after displacement can coexist with different degrees of skill and identity continuity, which we interpret as part of career reconstruction and acculturative adaptation.

These findings extend acculturation research by showing that adaptation after forced migration is not limited to language learning, social belonging or everyday cultural adjustment. It also involves learning how to navigate host-society labour-market expectations, legal procedures, workplace norms and community-based opportunity structures. Career reconstruction can therefore be understood as a domain-specific form of acculturative adaptation through which migrants negotiate continuity with prior occupational identities while rebuilding future direction within unequal social, economic and institutional conditions. More specifically, the study adds an occupational dimension to acculturation accounts by tracing how institutional access and skill recognition shape the possibilities for maintaining a professional identity, while social ties and perceived agency shape how migrants respond. These processes can coexist with employment: a person may find work yet remain unable to use prior qualifications or pursue a preferred occupation.

A central finding was that participants’ career reconstruction was constrained by limited access to reliable information and institutional guidance. Participants often lacked clear knowledge about education systems, legal procedures, credential recognition, employment routes and labour-market expectations. This aligns with research showing that migrants often struggle to access accurate information about legal status and support services, with consequences for career planning and employment decisions (Beqiraj & Ferrari, 2023). The findings also suggest that the problem was not only the absence of support but also the limited accessibility of existing services. Support programmes that require sustained attendance or do not account for caregiving, financial pressure and daily survival needs may remain formally available but practically inaccessible. In the absence of accessible support, participants relied on informal advice from relatives, acquaintances and local communities. Although these networks provided immediate assistance, they could not replace systematic, rights-based and culturally responsive guidance. From an ecological and systems-based perspective, migrants’ occupational choices were embedded within legal, linguistic, economic and institutional systems rather than being purely individual decisions (Bronfenbrenner, 1979; W. Patton & McMahon, 2014).

Language barriers further intensified these constraints. Consistent with previous studies, participants reported that limited proficiency in Turkish restricted access to information, education, formal employment and occupational continuity (Çirkin et al., 2025; Demir & Aliyev, 2019; Hokkinen & Barner-Rasmussen, 2023). For those who had previously worked as teachers, nurses, architects, drivers or business owners, language barriers did not merely create communication difficulties; they made prior knowledge and professional competence less visible in the host labour market. In this respect, language operated as a mechanism of occupational downgrading and deskilling, contributing to professional status loss and weakening participants’ ability to continue their pre-migration careers (Bauder, 2003; Guo, 2009; Hokkinen & Barner-Rasmussen, 2023). Language support should therefore be understood not only as a tool for social adaptation but also as a central condition for labour-market participation and occupational continuity.

Legal constraints and financial pressure also shaped participants’ career trajectories. Uncertainty around citizenship, work permissions, educational access, business establishment and credential recognition narrowed their perceived range of possibilities. These accounts should be read not only as descriptions of formal legal barriers but also as evidence of limited legal literacy and uncertainty about how rights, procedures and institutional routes operate in practice. Such uncertainty interacted with economic hardship, pushing participants toward immediate income-generating work rather than long-term career planning. This extends previous work on migrant labour-market integration by showing how legal precarity and financial pressure produce survival-oriented occupational transitions (Blustein, 2019; Sultana, 2022; Torlinska et al., 2020). Thus, career reconstruction after forced migration involved both agency and constraint: migrants made decisions, but within highly restricted opportunity structures.

The findings also revealed that career reconstruction after forced migration was gendered. Women in the study described how caregiving responsibilities, family expectations and culturally embedded gender roles limited their access to employment, training and occupational mobility. This aligns with intersectional approaches, which emphasise that migration status and gender jointly shape access to resources, rights and opportunities (Crenshaw, 1991; Zadhy & Erman, 2023). Women’s occupational choices were shaped not only by qualifications or aspirations but also by domestic responsibilities, childcare, mobility concerns and expectations to remain close to family. Rights-based support for displaced populations must therefore address not only legal and economic barriers but also the gendered organisation of everyday life. The participants’ accounts also show that gendered responsibilities interacted with available resources: P6 described childcare as a barrier to a training opportunity, whereas P9 described support from her daughters in her work. Given the small sample of employed participants, these accounts illuminate mechanisms within this study rather than establishing a population-level difference between women and men.

Another important finding concerns participants’ perceived agency. Some participants described their post-migration career trajectories as shaped by forces beyond their control, including war-related loss, legal uncertainty, financial hardship, age and restricted opportunities. Rather than interpreting these accounts as a stable external locus of control, they are better understood as expressions of constrained or bounded agency, in which individuals continue to act but within opportunity structures shaped by institutional, economic and social conditions (Evans, 2007). From the perspective of Social Cognitive Career Theory, perceived barriers may reduce career-related confidence and limit action, while supportive relationships, previous achievements and visible opportunities may strengthen self-efficacy and agency (Lent et al., 1994; Lent & Brown, 2013). In this study, agency therefore emerged not as context-free individual choice, but within limited spaces of action shaped by social support, information access, prior skills and available opportunities.

Within these constrained spaces, some participants engaged in occupational exploration and attempted to build new pathways. Exploration included observing others’ work, seeking advice, considering alternative occupations, pursuing education and planning future business activities. This aligns with Career Construction Theory, which emphasises that individuals seek continuity and meaning across changing life circumstances (Savickas, 2005, 2013). Yet exploration was not simply a career choice activity; it was a way of making sense of disruption, testing possibilities and rebuilding occupational direction under uncertainty. Transferable professional skills were central to this process. Participants often tried to rely on skills developed before migration, even when they could not continue in the same occupation. Prior skills helped maintain continuity between pre- and post-migration occupational identities, but they did not automatically produce career continuity. Their usefulness depended on language proficiency, legal recognition, local labour-market demand, social networks and institutional support, indicating that transferable skills functioned as potential resources whose activation required supportive social and institutional conditions (Bauder, 2003; Guo, 2009; Visintin et al., 2015).

Self-efficacy and future orientation also played important roles in participants’ career reconstruction. Participants who believed that they could learn, adapt or succeed in a new occupation were more likely to describe future goals and occupational plans. This is consistent with research linking self-efficacy to hope, adaptability and career-related action (Ginevra et al., 2021; Zhou & Kam, 2016). In this study, self-efficacy was closely connected to previous experience, observation of others and gradual familiarisation with new work contexts. Future orientation helped participants interpret occupational change not only as loss but also as a possible route toward renewed meaning. Together, self-efficacy, exploration and future orientation operated as interrelated components of adaptive career reconstruction, enabling displaced individuals to cope with disruption while imagining possible futures within constrained conditions.

Across these processes, informal social support emerged as the most visible and frequently used resource. Participants relied on relatives, friends, local residents and migrant community networks for housing, information, job access, emotional security and occupational direction. Previous research similarly shows that social support can strengthen migrants’ coping strategies, labour-market access and social participation (Fejes et al., 2022; Yang et al., 2023; Zhang et al., 2023). The findings also resonate with Social Identity Theory and Self-Categorisation Theory, which suggest that shared group membership can shape belonging, perceived safety and access to resources (Tajfel & Turner, 1979; Turner et al., 1987). Arabic-speaking and culturally familiar spaces functioned as short-term buffer zones against exclusion, but they could also reproduce bounded labour-market opportunities when they reduced exposure to Turkish-language networks, formal institutions and wider employment routes.

The importance of informal social support should therefore not obscure the need to reduce structural barriers. When social networks become the main substitute for formal guidance, migrants’ opportunities may remain confined to socially bounded routes into work. These networks may help individuals find immediate employment, but they may also limit access to formal training, credential recognition processes and more diverse occupational pathways. Effective career reconstruction after forced migration requires both strengthened community-based support and accessible institutional mechanisms that expand rather than narrow migrants’ occupational possibilities.

Career reconstruction also depends on how members of the host society respond to migrants. Acculturation is a reciprocal process in which host-society attitudes and relationships shape the options available to newcomers (Berry, 1997; Bourhis et al., 1997). Domínguez and Maya-Jariego (2008) found that host individuals often occupy peripheral positions in immigrants’ personal networks, becoming more central with longer residence, and that some, such as bilingual service providers, act as brokers connecting migrants with local resources. Several participants’ accounts point to such host-side roles: P1 opened his shop in a room provided by a local benefactor, and P9 described not feeling like a stranger in Şanlıurfa, where part of the local population speaks Arabic. In Türkiye, public attitudes towards Syrians under temporary protection also vary across occupational groups and are least inclusive in contexts such as business partnerships and renting homes (Kasımoğlu & Taşdan, 2026), which bear directly on migrants’ efforts to open businesses or move to other cities. Access to host-society brokers and the attitudes of employers, landlords and co-workers may therefore shape which opportunities become available to migrants with similar personal resources. Because the present study relied on migrants’ accounts, future research could include employers, co-workers and service providers to examine directly how host-society attitudes shape career reconstruction.

The central contribution of this study is to show that forced migrants’ occupational trajectories cannot be adequately understood through employment status alone. They involve a negotiated process of career reconstruction in which legal constraints, language barriers, financial pressure, gendered responsibilities, social networks, prior skills and perceived agency jointly shape migrants’ capacity to rebuild working lives. The findings also show that personal and social resources were unevenly distributed and could not be understood as individual assets alone; their activation depended on gender, education, previous occupation, language proficiency and access to supportive networks. The findings therefore connect career reconstruction, labour-market integration, sociocultural adaptation and structural inequality within the context of Syrian migrants in Türkiye.

### 4.1. Implications for Practice and Policy

The findings have implications for both career support practice and migration-related policy. Support for forcibly displaced people should move beyond short-term job placement and address the broader conditions shaping labour-market integration. Rights-based and culturally responsive services should combine vocational guidance with legal orientation, language support, recognition of prior learning and individualised skill mapping. Such services can help migrants identify transferable competencies, rebuild occupational identities and develop realistic but future-oriented plans within the constraints of the host context (Arthur et al., 2025; Blustein, 2019; Sultana, 2022).

The findings also suggest that informal community support should be connected more effectively with formal institutions. Social networks provided participants with belonging, information and practical assistance, but reliance on these networks alone may confine migrants to limited labour-market opportunities. Community-based career centres, peer mentoring, bilingual career navigation and partnerships among migration authorities, educational institutions, employment agencies and employers may help displaced individuals access broader and more sustainable occupational pathways (Fejes et al., 2022; Ryan et al., 2008).

Policy and practice should also address gender-specific barriers, as migration status and gender jointly shape access to work, training and mobility (Crenshaw, 1991; Zadhy & Erman, 2023). For women migrants, childcare-sensitive schedules, flexible training models and family-inclusive counselling where appropriate may reduce barriers related to care responsibilities, family expectations and mobility constraints. Practitioners working with forcibly displaced populations also need competencies beyond traditional career guidance, including trauma-informed communication, cultural humility, awareness of migration-related legal procedures, sensitivity to structural inequality and the ability to integrate psychosocial support with occupational planning. Training programmes for career practitioners should therefore include content on forced migration, sociocultural adaptation, rights-based guidance and career reconstruction.

### 4.2. Limitations and Future Research

This study has several limitations. First, it was conducted with a relatively small sample of Syrian migrants living in specific regions of Türkiye. The interviews provided context-specific accounts of participants’ experiences; however, the findings should be interpreted as exploratory and considered in relation to Türkiye’s specific legal, linguistic, cultural and labour-market context. Second, the study was cross-sectional and based on retrospective accounts; therefore, it could not capture how migrants’ working lives, support needs and perceptions of agency change across different stages of acculturative adaptation. Future longitudinal studies may examine how career reconstruction unfolds over time as migrants acquire language proficiency, expand social networks, encounter institutional systems and renegotiate occupational identities. One interview lasted approximately 25 min, which may have restricted the depth of that participant’s account relative to the other nine interviews of approximately 40–45 min. Recruitment and the initial interviews were challenging in part because concerns related to legal status made some individuals hesitant to discuss their migration and employment experiences. In-depth follow-up interviews with all participants were not feasible. Only two participants took part in brief telephone validation interviews; of the other eight, five could not be reached and three declined a further interview. The study therefore relies mainly on one in-depth interview per participant and had limited opportunity to revisit emerging issues or deepen accounts through repeated contact.

Third, all participants had participated in working life both before and after migration. The study therefore does not represent forcibly displaced individuals who remain unemployed, economically inactive or unable to work because of health, caregiving, legal or social barriers. Although the sample included variation in gender, age, education and occupational background, larger and more diverse qualitative studies could further examine how these characteristics shape career reconstruction among employed, unemployed and economically inactive forced migrants. Comparative studies across different host countries, legal statuses and migration histories may also clarify how social and psychological determinants of acculturation operate under different institutional and labour-market conditions.

## 5. Conclusions

This study conceptualised career reconstruction as a domain-specific form of acculturative adaptation among Syrian migrants in Türkiye. Within these ten accounts, participants described disruptions to occupational continuity and professional identity amid restrictive legal, linguistic, economic, institutional and sociocultural conditions. Limited institutional guidance, language barriers, legal constraints, financial pressure and gendered responsibilities narrowed occupational possibilities and often pushed participants toward survival-oriented work. At the same time, informal social support, perceived agency, self-efficacy, future orientation, occupational exploration and transferable professional skills helped participants create limited spaces of action within these constraints.

The study contributes to acculturation and migration scholarship by showing that working life is a central but underexamined domain in which social and psychological adaptation takes place after forced migration. Career reconstruction was not simply a matter of employment, nor was it only an individual response to displacement. Rather, it emerged as a negotiated process through which migrants attempted to rebuild occupational continuity, professional meaning, belonging and future direction in the host-society context. Supporting this process requires more than job placement; it requires rights-based, culturally responsive and community-linked services that combine language support, legal orientation, recognition of prior learning, gender-sensitive provision and accessible career guidance. Career reconstruction should therefore be understood as an ongoing and contingent process, as migrants’ opportunities, responsibilities and occupational aspirations change over time. Longitudinal research could examine how these negotiations evolve with changes in language proficiency, family circumstances, institutional access and work experience.

## Figures and Tables

**Table 1:** Participants’ sociodemographic and occupational characteristics.

No.	Gender	Age	Educational Background	Occupation Before Migration	Occupation After Migration	Year of Migration	Marital Status
P1	Male	32	Bachelor’s degree	Teacher	Barber	2015	Married
P2	Male	27	High school	Construction worker	Farmer	2013	Married
P3	Male	40	No formal education	Excavator operator	Tractor driver	2014	Married
P4	Female	29	Bachelor’s degree	Architect	Accountant	2016	Single
P5	Female	52	Associate degree	Preschool teacher	Cleaner	2015	Married
P6	Female	56	Bachelor’s degree	Nurse	Tailor	2016	Married
P7	Female	33	Bachelor’s degree	English teacher	Translator	2015	Married
P8	Male	38	High school	Plasterer	Animal caretaker	2014	Married
P9	Female	45	No formal education	Grocery store owner	Livestock trader	2014	Married
P10	Male	45	Primary school	Bus driver	Farmer	2013	Married

## Data Availability

The data presented in this study are not publicly available due to confidentiality and ethical restrictions related to the sensitive nature of forced migration experiences. Anonymized excerpts relevant to the analysis are included in the article.
